# A THEORETICAL FRAMEWORK TO UNDERSTAND INTRINSIC HEALTH

**DOI:** 10.1093/geroni/igad104.0115

**Published:** 2023-12-21

**Authors:** Alan Cohen, Martin Picard, John Beard, Daniel Belsky, Linda Fried, Kelli Hall, Julie Herbstman, Nour Makarem

**Affiliations:** Columbia University, New York City, New York, United States; Columbia University Irving Medical Center, New York City, New York, United States; Columbia University, New York City, New York, United States; Columbia University, New York City, New York, United States; Columbia University, New York City, New York, United States; Columbia University, New York City, New York, United States; Columbia University, New York City, New York, United States; Columbia University, New York City, New York, United States

## Abstract

Traditional approaches to measuring health have relied largely on measures of function and capacity, or on subjective assessments. These approaches are important, but they result in a large gap between the biology underlying health and our ability to measure it. Here, we propose a novel concept – intrinsic health – defined as a field-like state emerging from the dynamic interplay of energy, communication, and structure within the organism, which enables robustness, resilience, adaptability, and performance. It reflects the capacity of an organism to maintain its internal dynamic equilibrium. These three pillars – energy, communication, and structure – are derived from first principles, and are the basic ingredients of life. Disruption or optimization in the organized flow of energy and information through biological structures provide a foundation to understand the causes of disease, and the foundation for health, respectively. For each pillar, we discuss available measurements that cover multiple levels of biological organization, empirical evidence linking them to traditional health outcomes, and also the limitations of isolated measurements. This motivates the use of more integrative, time-series data reflecting the dynamic psychobiological interplay among organ systems. We then situate intrinsic health in the context of environmental, behavioral, and cultural factors that affect how it is transformed into “realized health,” the health we experience as individuals and populations. The intrinsic health framework can be extended to understand how intrinsic health intersects with aging, disease, and wounds. Finally, we propose a measurement framework to quantify intrinsic health at the individual level.

